# Supplementary material for: Wind-driven device for cooling permafrost
Source: Nat Commun. 2023 Nov 20;14:7558. doi: 10.1038/s41467-023-43375-z (PMC10662160; doi:10.1038/s41467-023-43375-z)
Supplement: Supplementary file 1 — Supplementary Information [file 41467_2023_43375_MOESM1_ESM.pdf]

Supplementary Information

Wind-driven device for cooling permafrost

Yinghong Qin [1,2], Tianyu Wang [2,*], Weixin Yuan [2]

1. School of Civil Engineering and Architecture, Guangxi Minzu University, 188 University Road, Nanning 530006, China

2. College of Civil Engineering and Architecture, Guangxi University, 100 University Road, Nanning 530004, China

* Correspondence: tywang@st.gxu.edu.cn (T. Wang)

The air temperature duration during the experiment

In our comprehensive dataset spanning from September 1, 2020, to September 1, 2022, we observed a diverse range of air temperatures, reflecting the intricate climatic variations inherent to the study region (Fig. S1). The mean air temperature over this duration was recorded at -2.42 °C, with a median value of -1.74 °C, underscoring the prevalent cooler conditions. We set the freezing temperature of the solid-liquid phase change material in the clutch to -4 °C. Consequently, the operational duration of the device is approximately 0.288 of the year. If the freezing temperature is set a higher level, such as -2 °C, the operational time of the device will increase. In the future study, we will further investigate the optimal freezing temperature of the clutch to maximize the cooling capacity. Further studies are also expected to understand if the efficiency of the cooling process and the clutch freezing temperature are site-dependent.

Notably, the measurement captured extreme temperature fluctuations, with a minimum temperature plummeting to a stark -21.48 °C and a maximum reaching 13.14 °C. The standard deviation of 6.05 °C further elucidates the pronounced variability in the temperature readings, emphasizing the need for meticulous consideration of such variations in related environmental and climatic studies.

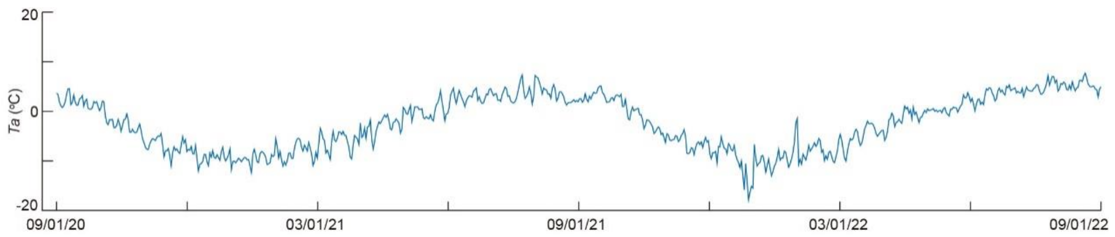

Fig. S1 Daily Mean Air Temperature Trends from September 1, 2020, to September 1, 2022 illustrates both the seasonal variations and transient temperature anomalies over the two-year period. Source data are provided as a Source Data file.

The wind speed during the experiment

Upon meticulous examination of the daily wind speed data from September 1, 2020, to September 1, 2022, intricate patterns of atmospheric dynamics were unveiled (Fig. S2a). The dataset reached a peak daily mean wind speed of approximately 11.94 m/s, a testament to the occasionally intense wind conditions of the study region. Furthermore, the observed standard deviation of approximately 1.75 m/s in daily wind speeds underscores the variability and unpredictability of short-term wind behaviors, necessitating robust adaptive strategies for applications reliant on consistent wind patterns.

Delving into the monthly aggregated wind speed data, a pronounced seasonality emerges, with winter months consistently registering elevated wind speeds relative to the more temperate summer months. The dataset reveals a peak monthly mean wind speed of approximately 5.80 m/s, juxtaposed against a minimum of 2.65 m/s, highlighting the range of wind conditions experienced over the year (Fig. S2b). The observed standard deviation of approximately 0.83 m/s further underscores the variability in monthly wind speeds. This seasonally heightened wind activity, particularly during the colder months, not only accentuates the potential for increased energy yield but also emphasizes the significance of infrastructural adaptability. The consistent and robust wind speeds during winter present an opportune window for harnessing this renewable resource to circulate the liquid coolant to carry heat in the permafrost stratum to the air during periods of heightened wind activity.

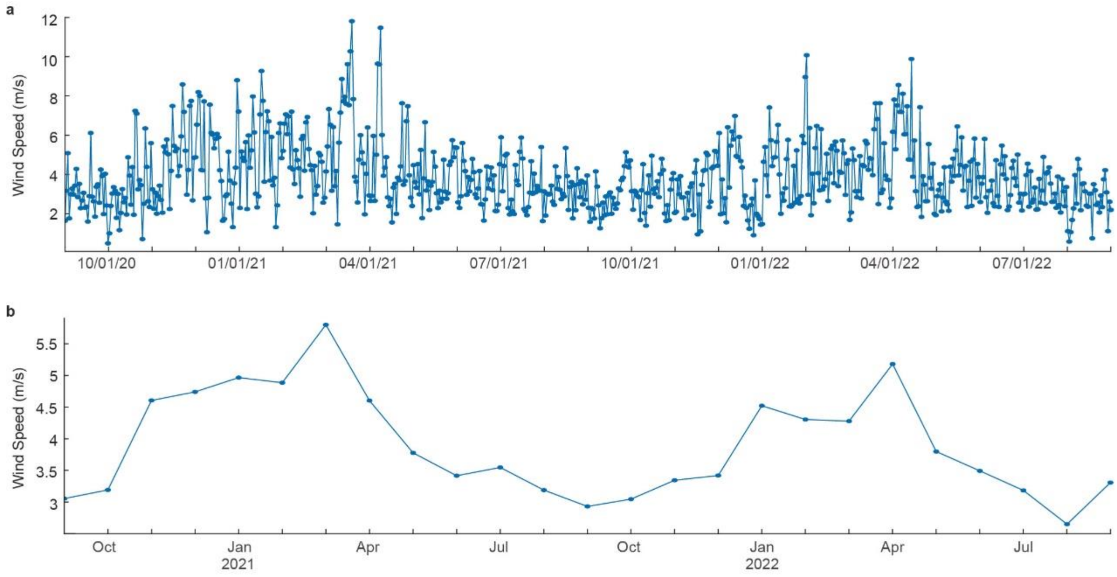

Fig. S2 Temporal analysis of wind speeds. **a** Daily mean wind speed from September 1, 2020, to September 1, 2022, highlighting the variability and peak wind conditions. **b** Monthly mean wind speed over the same period, emphasizing the pronounced seasonality with elevated wind speeds during winter months compared to summer. Source data are provided as a Source Data file.

The correlation between the wind speed and the pump discharge rate

We meticulously examined the interplay between wind speed (WS) and the discharge rate of a pump (v) utilized in the fluid-circulation heat exchanger. The experimental setup was strategically positioned within a wind tunnel, where the discharge of the circulating coolant was monitored across a wind speed spectrum ranging from 0 to 12 m/s. A wind cup, placed 1m ahead of the windmill, facilitated these observations. Each specific wind speed was maintained for a duration of 20 minutes, post which the discharge volume was quantified by weight. Notably, wind speeds below 1.8 m/s rendered the windmill static, resulting in a discharge rate of 0 ml/s. Conversely, wind speeds exceeding 1.8 m/s activated the windmill, leading to a linear increase in discharge volume in tandem with the wind speed. A rigorous regression analysis revealed a compelling linear relationship between wind speed and discharge rate, described by the equation $v=21.16\times(\mathrm{WS}-1.77)$, boasting an R-squared value of 0.94 (Fig. S3).

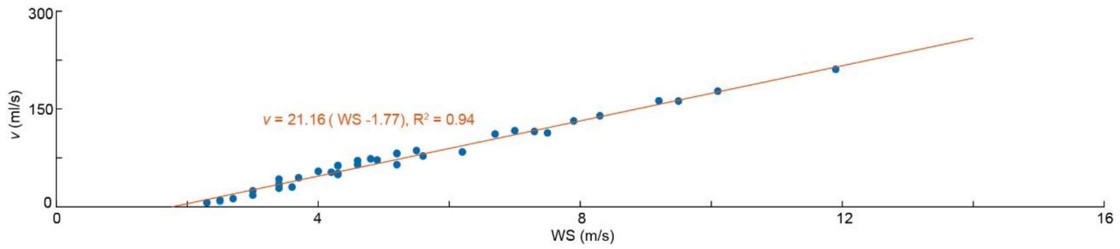

Fig. S3 The pump’s discharge rate increases linearly with the wind speed. Source data are provided as a Source Data file.

The discharge rate of the pump during the experiment

In our field-based investigations, quantifying the discharge of the liquid coolant within the fluid-circulation heat exchanger presented significant challenges, primarily due to the intricacies associated with integrating a flowmeter within the device. Consequently, we adopted an indirect approach to ascertain the device's discharge rate. By leveraging the established correlation between wind speed and the pump's discharge rate, and incorporating the empirically measured wind speeds, we derived the discharge rate of the device. This methodology culminated in the results depicted in Fig. S4.

Our meticulous observations of the daily mean discharge rate revealed a pronounced maximum of approximately 148.39 mL/s. This peak, juxtaposed with the minimum of 0 mL/s, underscores the inherent variability in daily discharge rates, with an average rate of around 20.58 mL/s. A salient observation was that, at certain instances, particularly near the maximum discharge rate, the flow might be exceedingly rapid, potentially curtailing the requisite time for effective heat transfer from the surrounding soil to the coolant. Such rapid discharge rates could impede optimal energy transfer, warranting careful consideration in system design and operation.

A seasonal analysis of the monthly mean discharge rate, with an average of approximately 20.72 mL/s, revealed a distinct dormancy during the summer months, where the discharge rate consistently registered at 0 mL/s. The peak monthly rate was observed to be around 70.82 mL/s. This hibernation phenomenon is indicative of the device's adaptive response to ambient conditions. However, it's imperative to note

that the discharge rate's magnitude and variability, ranging from 0 mL/s to 70.82 mL/s, are significantly influenced by the pump's parameters. The observed variations in discharge rates, especially during active months, suggest that the pump's specifications play a pivotal role in the device's performance. As we delve deeper into understanding the cooling capabilities of the device, future investigations will focus on evaluating its efficacy with pumps of varying parameters.

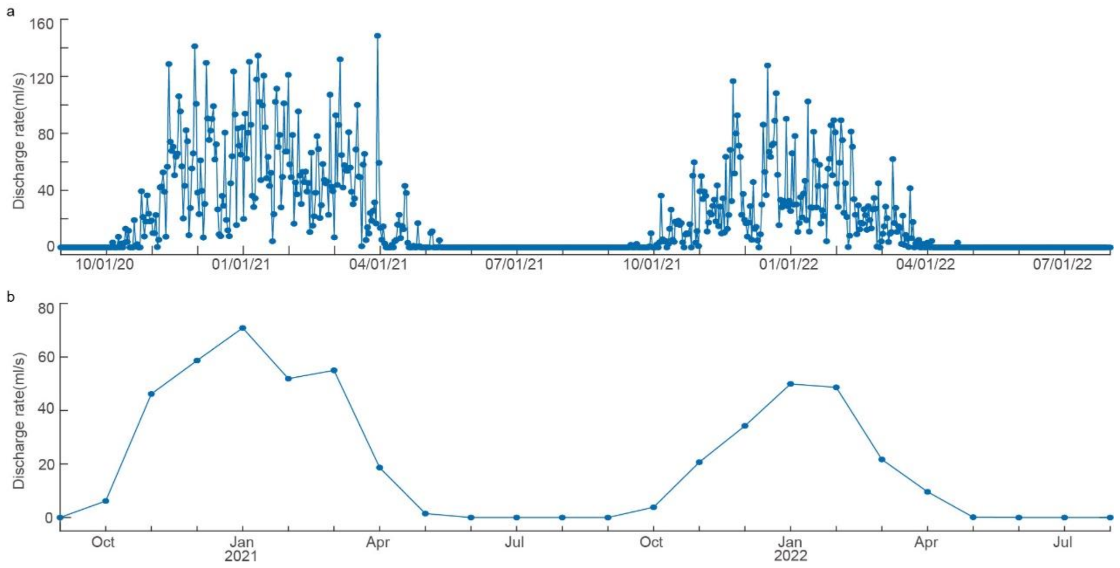

Fig. S4 Temporal variation of pump discharge rate. **a** The daily mean discharge rate over time. **b** The monthly mean discharge rate. Notable is the device's adaptive response to ambient conditions, with periods of heightened activity interspersed with dormant phases. Source data are provided as a Source Data file.

The power of the device

Getting the discharge rate, one can compute the power of the device using

$$p = c\rho v\Delta T \qquad \text{(S-1)}$$

where for the coolant c = 3121 J·g^{-1}·K^{-1}, ρ = 1058 kg/m^3. ΔT represents the temperature gradient between the coolant at the device's base and the peak temperature of the upward flow extending from the base to the ground surface. For illustrative purposes, this differential can be derived from the data presented in Fig. 3a-i. Employing this computational methodology, we discerned the temperature

gradient during clutch engagement. As depicted in Fig. S5a, the temperature disparity, ΔT, oscillates around a mean of 0.8 °C. Integrating this temperature gradient into Eq. (S-1) facilitates the determination of the device's power output. A scrutiny of Fig. S5a reveals peak instantaneous power outputs reaching an impressive 300 W. An annualized assessment suggests the device's potential to consistently deliver an average power of 68.03 W. It's imperative to acknowledge that such power metrics are susceptible to variations influenced by the phase change material's (PCM) freezing temperature within the clutch, the pump's operational parameters, and prevailing ambient air temperatures.

In our comparative analysis, we juxtaposed the power output of our device with that of a thermosyphon, as detailed by Zhang *et al.* [1]. Zhang and colleagues elucidate the nuances of thermosyphon power, underscoring its dependence on the temperature gradient between the evaporator and condenser—a principle consistent with our investigative approach. By imposing a temperature boundary condition at the ground surface to emulate thermosyphon temperature, they propose a power equation $p=k\times(T_e-T_c)$, where k is a constant, and T_e and T_c are the temperatures of the evaporator and condenser, respectively. Their derived power spans a range of 0 to 120W, averaging an annual power of 30 W (Fig. S5c). It's crucial to underscore that this 30 W measure stems from computational modeling. In a recent experiment study by the same research team [2], the thermosyphon's power during active phases was recorded below 25 W (Fig. S5d). By incorporating durations of thermosyphon inactivity, the projected annual power is discernibly less than 25 W. Similarly, the thermosyphon's efficiency, influenced by parameters such as fluid selection, design intricacies, and operational conditions, critically dictates its power potential. A discerning examination of Fig. S5b juxtaposed with Fig. S5c-d reveals the superior efficacy of our wind-driven cooling device over the conventional thermosyphon.

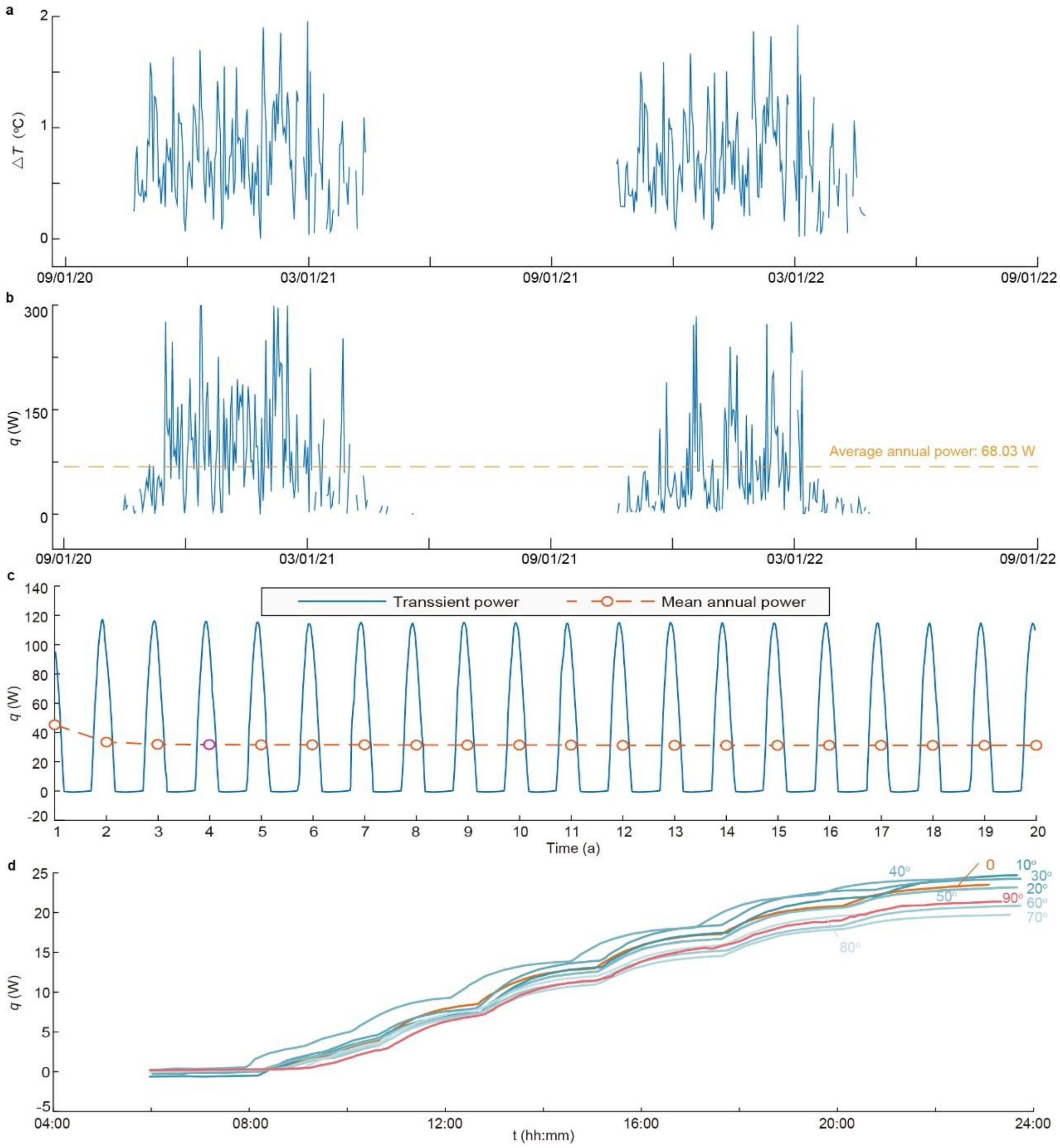

Fig. S5 The power of the wind-driven cooling device and of the thermopython. **a** The temperatures increase along the outer annulus of the tube. **b** The power of the device varies over time. **c-d** The power of the thermosypon estimated by Zhang *et al.* (Reprinted from references [1, 2], Copyright (2017, 2022), with permission from Elsevier).

Reference:

1. Zhang M, Pei W, Lai Y, Niu F, Li S. Numerical study of the thermal characteristics of a shallow tunnel section with a two-phase closed thermosyphon group in a permafrost region under climate warming. *Int J Heat*

Mass Transfer **104**, 952-963 (2017).

2. Zhang M, Yan Z, Pei W, Lai Y, Qin Y, Yu F. Experimental study on the startup and heat transfer behaviors of a two-phase closed thermosyphon at subzero temperatures. *Int J Heat Mass Transfer* **190**, 122283 (2022).
